# Neuraminidase activity and specificity of influenza A virus are influenced by haemagglutinin-receptor binding

**DOI:** 10.1080/22221751.2019.1581034

**Published:** 2019-02-27

**Authors:** Jimmy Chun Cheong Lai, Herath M. T. K. Karunarathna, Ho Him Wong, Joseph S. M. Peiris, John M. Nicholls

**Affiliations:** a Department of Pathology, The University of Hong Kong, Hong Kong, Hong Kong SAR; b HKU-Pasteur Research Pole, The University of Hong Kong, Hong Kong, Hong Kong SAR; c School of Public Health, The University of Hong Kong, Hong Kong, Hong Kong SAR; d Department of Veterinary Public Health and Pharmacology, Faculty of Veterinary Medicine and Animal Science, The University of Peradeniya, Peradeniya, Sri Lanka

**Keywords:** Influenza virus, neuraminidase, Hemagglutinin, receptor interaction, sialic acids

## Abstract

Influenza virus haemagglutinin (HA) and neuraminidase (NA) are involved in the recognition and modulation of sialic acids on the cell surface as the virus receptor. Although the balance between two proteins functions has been found to be crucial for viral fitness, the interplay between the proteins has not been well established. Herein we present evidence for interplay between influenza HA and NA, which may affect the balance between two glycoprotein functions. NA enzymatic activities against sialoglycans were promoted by the presence of HA, which is in accordance with the level of co-existing HA. Such activity enhancement was lost when the HA-receptor binding properties were abolished by low-pH treatment or by mutations at the HA receptor binding domain. Sialidase activities of NA-containing virus-like particles and native influenza viruses were detected using different NA-assays and sialic acid substrates. Most pronounced HA-mediated NA enhancement was found when intact virions were confronted with multivalent surface-anchored substrates, which mimics the physiological conditions on cell membranes. Using recombinant viruses with altered HA bindings preference between α2,3- and α2,6-linked sialic acids, we also found that NA function against different substrates is correlated with the HA-receptor specificity. The effect of HA-receptor specificities on NA functions, together with the HA-mediated NA enhancement, may play a role in virus evasion of the mucus barrier, as well as in cross-species adaptation. Our data also indicate the importance of using multivalent substrates in future studies of NA functions.

## Introduction

Influenza A viruses continue to circulate in humans and in various animal species including birds, pigs, horses and dogs [1]. The annual epidemics of seasonal influenza are responsible for millions of human infections worldwide leading to significant morbidity, mortality and economic burden [2]. Human influenza pandemics arise when novel subtypes of influenza viruses emerge from animals through genetic reassortment and in the past these spread within months across the world, leading to large numbers of deaths. Two major surface glycoproteins of influenza viruses, the haemagglutinin (HA) and neuraminidase (NA), both recognize the host-cell sialic acid (SA) molecules as the influenza virus receptor [3]. A functional balance between HA and NA has been proposed to be a major determinant for successful viral replication and fitness [4].

Influenza HA is a trimeric protein which binds to host cell SA-receptors as the initial step of virus attachment [5,6]. Fusion between virus and host-cell endosomal membranes is also mediated by HA [6,7]. Upon virus entry into host cell via endocytosis, the acidic environment of the endosome induces a conformational change in HA that exposes the fusion peptide, allowing for viral-endosomal fusion. Neuraminidases are expressed on the viral surface as tetramers and serve to promote the release of progeny viruses from the infected cells as well as the penetration through host mucus by desialylation of viral and cellular surface glycans [8,9]. NA also plays a role in the initial stage of virus infection by facilitating virus motility on the cell surface [10].

In HA-receptor bindings, sialoglycans with α2,3-linked SA are generally regarded as avian-like receptors, while those contain α2,6 SA linkage are considered as human-like receptors. Whereas conserved NA specificity against α2,3-linked SA was found among viruses from different hosts [11,12], virus isolates with HA specificity towards human-like receptors are usually associated with an increase in NA activity against α2,6-linked SA [13,14]. The balance of HA and NA, in reassortant viruses from different hosts or in the deficiency of NA activity, can be restored by an adjustment of receptor specificities [15] or a compensatory decrease of receptor binding affinity of the HA [16,17]. Since both glycoproteins target cell surface SA molecules, it is likely that HA and NA functionally interact with each other. It was previously demonstrated that cooperation between HA and NA contributes to the viral motility on the receptor-coated surface [18,19]. Another study showed that virion NA activity in the enzyme-linked lectin assay (ELLA) was inhibited by anti-HA antibodies [20], suggesting that virus binding to immobilized fetuin may enhance NA activity. However, experimental evidence for the influence between the functions of the two glycoproteins remains very limited.

We have previously generated influenza virus-like particles (VLPs) for the study of HA and NA functions [21,22]. In this study, we investigated the effect of HA-receptor binding on the modulation of NA activity in influenza virions as well as in NA-containing influenza VLPs. With a modulation of the pH to selectively impede HA binding, but retaining NA activity, we examined the functional interaction between HA and NA in virus preparations. The effect of altered HA-receptor preference on the substrate specificity of NA was also studied. Overall, our data suggested that enzyme kinetics and substrate specificity of influenza neuraminidase are influenced by HA binding to SA-receptors.

## Results

### Differential NA activity on native influenza virus and NA-expressing VLPs

**Figure 1.**
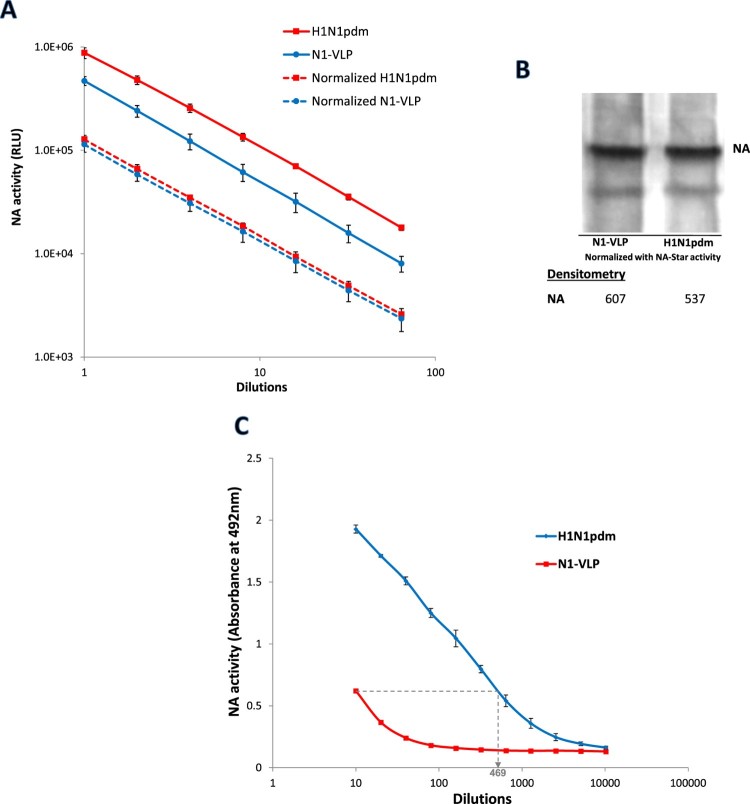
Differential NA activities from H1N1pdm influenza virus and NA-VLP. (A) NA activity of H1N1pdm virus and N1-VLP was detected and normalized using NA-Star kit. The NA activities were measured in Relative Luminescence Units (RLU) and plotted against dilutions. (B) The levels of NA protein (75 and 55 kDa) in the normalized H1N1pdm virus and N1-VLP samples were detected by western blotting using rabbit anti-NA antisera. (C) NA activities of the normalized samples against fetuin were determined in Enzyme-Linked-Lectin-Assay (ELLA). Desialylation of fetuin were detected using HRP-conjugated PNA lectin followed by OPD. Mean absorbance at 492 nm is plotted against sample dilutions. The experiments have been repeated twice with similar results.

VLPs containing NA from H1N1pdm influenza virus were constructed by overexpression of NA in mammalian cells as previously described [23,24]. Enzymatic activities of N1-VLP and native H1N1pdm viruses were measured by serial dilutions in an NA-Star assay, and the two preparations were normalized for their cleavage activities against the NA-Star substrate (Figure 1(A)). Two glycosylated forms of NA at ∼75 and ∼55 kDa were detected in western blotting as reported [25] and NA protein levels of the normalized samples were found to be similar (Figure 1(B)). However, with normalized NA contents, the sialidase activity of the H1N1pdm virus was 46.9 folds higher than that of the N1-VLP when sialic acids cleavage of fetuin was detected in ELLA (Figure 1(C)). N1-VLP appeared to have markedly lower SA cleavage activity against immobilized fetuin when compared with that against chemiluminescent NA-Star substrate. As NA from the same virus strain was expressed on both N1-VLPs and H1N1pdm virions, it is likely that NA activity was enhanced by another viral component on the influenza virus. Although numerous viral components were missing in N1-VLPs, only the surface HA is known to interact with sialic acids and, therefore, it is rational that the concurrent expression of HA and NA facilitates the cleavage activity of NA against fetuin.

### Addition of influenza HA to NA-VLPs enhances the enzymatic activity in ELLA

**Figure 2.**
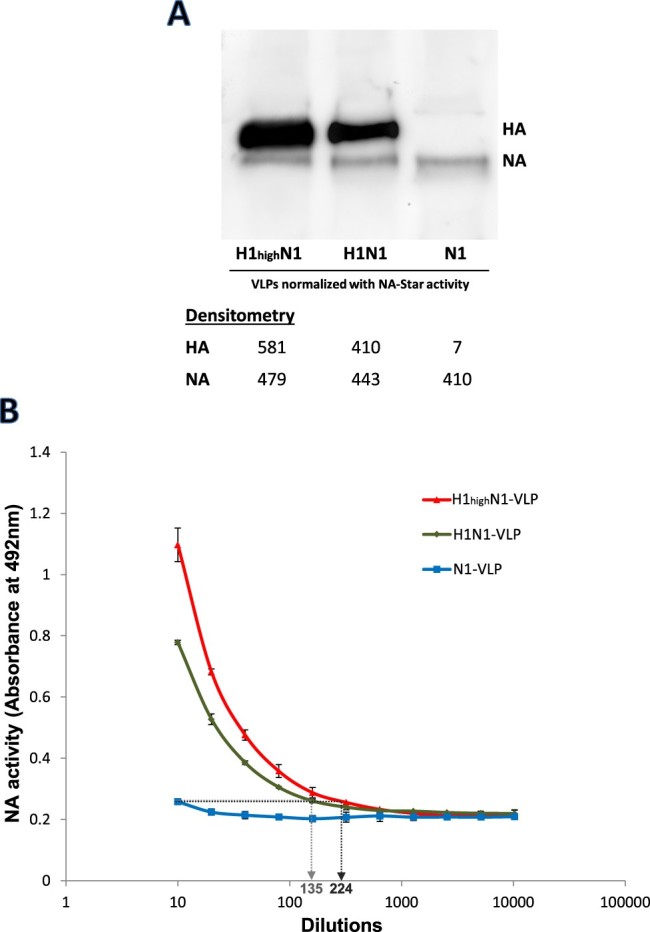
NA activities of NA-VLPs co-expressed with different levels of HA.H1N1-VLPs with different HA levels were collected from 293 T cells transfected with various DNA plasmids ratio and were normalized by the activities in NA-Star assays. (A) FLAG-tagged HA and NA protein levels (80 and 75 kDa) of the normalized VLPs were determined by western blotting using anti-FLAG monoclonal antibody. (B) Desialylation of fetuin by the VLPs was determined in ELLA. Mean absorbance at 492 nm is plotted against sample.

The effect of HA-NA co-expression on NA activity was investigated by the addition of HA to NA-VLPs during production. VLPs expressing various levels of FLAG-tagged HA and NA from H1N1pdm influenza (N1-VLP, H1N1-VLP and H1_high_N1-VLP) were prepared by adjusting DNA ratios during transfection. The VLPs were normalized by their NA activities in NA-Star assay as mentioned above (Figure S1), and western blotting analysis using anti-FLAG antibody reflected a similar level of NA proteins with varied HA contents (Figure 2(A)). Despite the samples being normalized for comparable NA content and activity on NA-Star assay, elevated NA activity against immobilized fetuin in ELLA was detected in VLPs with higher HA content. The increments were 13.5 folds in H1N1-VLPs and 22.4 folds in H1_high_N1-VLPs compared to N1-VLPs without HA expression (Figure 2(B)), indicating that NA functionality is enhanced in accordance with the level of co-expressed HA. However, ELLA activities of the HA/NA co-expressing VLPS could not reach a similar level as the native virus, probably due to a difference in glycoproteins density between VLPs and whole virions.

### Haemagglutinin-receptor binding was abolished by low pH treatment

**Figure 3.**
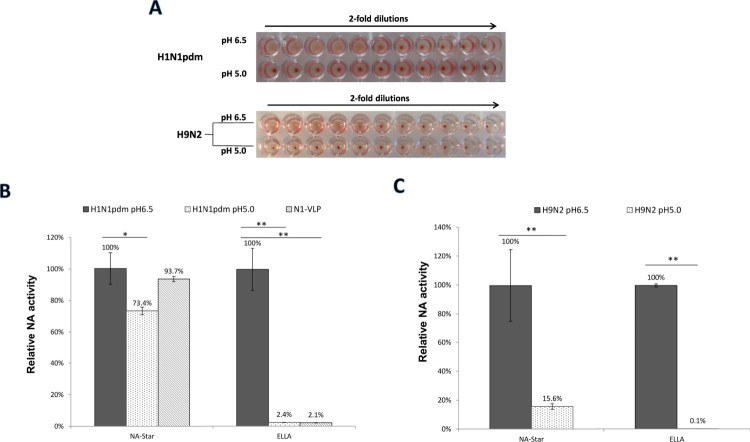
Effect of low pH treatment on HA and NA functions of influenza viruses.(A) Human H1N1pdm viruses and avian H9N2 influenza viruses were treated with acetate buffer at pH 6.5 or pH 5.0 and HA-receptor bindings were tested by haemagglutination of Turkey red blood cells in 2-fold dilutions. NA activities of the treated viruses (B) H1N1pdm and (C) avian H9N2 were measured by NA-Star assay or ELLA. Data from N1-VLP was included for comparison with the native H1N1pdm virus. Relative NA activities were calculated using the serial dilution curves obtained from the viruses. Mean values of 3 independent experiments were showed. *p < .05; **p < .01.

Influenza HA is sensitive to low pH which leads to conformational changes that expose the amino terminus of HA2 that mediates membrane fusion [26]. Influenza viruses exposed to acidic pH have been reported to cause rapid inactivation of the viral infection [27,28]. To assess whether we could selectively modulate the functions of HA in whole virions by inducing the conformational change, the effect of low pH on HA-mediated haemagglutination was tested. Human H1N1pdm and avian H9N2 (HK/G1/97) influenza viruses were pre-treated with TPCK-trypsin followed by incubation in acetate buffer (at pH 5.0 and 6.5). SA-binding properties of the treated viruses were determined by agglutination of Turkey red blood cells (TRBC) (Figure 3(A)). The ability to agglutinate TRBC was lost in both H1N1pdm and H9N2 viruses after pre-incubation at pH 5.0, compared to the control treatment at pH 6.5, indicating a loss of HA-receptor-binding property.

### Enhancement of NA activity by HA is dependent on the sialic acid-binding properties

The effect of HA-receptor binding on the level of NA activity was investigated by low pH treatment on influenza viruses. Compared to the control treatment at pH 6.5, H1N1pdm viruses pre-treated at pH 5.0 showed a decrease (26.6%) of NA activity in NA-Star assay, but a marked reduction (97.6%) was detected using ELLA (Figure 3(B)). The NA of the H9N2 virus displayed a lower tolerance to pH 5.0 with the NA activity in NA-Star assay dropped to 15.6% from that in the control treatment, but a dramatic reduction (99.9% or 716-folds decrease) was detected using ELLA assay (Figure 3(C)).

**Figure 4.**
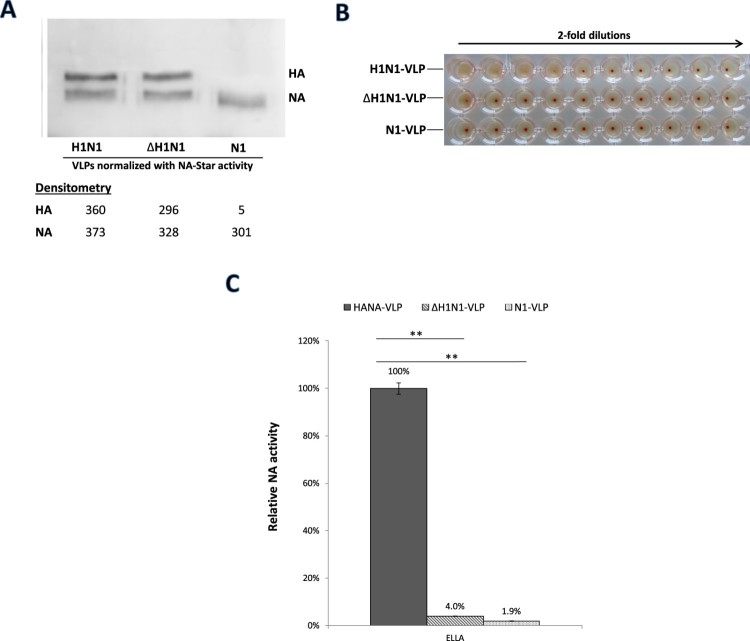
HA and NA functions of HANA-VLPs with deficient RBS mutation in HA. H1N1-VLPs expressing NA with either wildtype HA or ΔHA mutant (L194AY195F) from H1N1pdm were constructed and normalized with the activities in NA-Star assay. (A) HA and NA protein levels of the normalized samples were checked by western blot. (B) HA-receptor bindings were tested by haemagglutination of Turkey red blood cells in 2-fold dilutions. (C) NA activities of the VLPs against fetuin were determined in ELLA. Data obtained from N1-VLPs without HA co-expression was included for comparison. Relative NA activities were calculated using the serial dilution curves. Mean values of 3 independent experiments were showed. *p < .05; **p < .01.

As an alternative approach to abolish HA binding to SA receptors, influenza HA with amino acid mutations at positions 194 and 195 in the receptor-binding site (RBS) was generated (ΔH1N1-VLPs). These sites were reported to be crucial for receptor binding [29,30]. N1-VLPs, H1N1-VLPs and ΔH1N1-VLPs were normalized by their NA-Star activities (Figure S1). HA protein levels were checked in SDS-PAGE with densitometry analysis, showing that the expression of the HA mutant is slightly lower than the wildtype HA (Figure 4(A)). ΔH1N1-VLPs with the HA-defective mutations were unable to agglutinate TRBC, while the VLPs with the parental HA (H1N1-VLPs) had a HA titre of 16 (Figure 4(B)). Haemagglutination of TRBC was not observed in N1-VLPs without HA expression as expected. H1N1-VLPs were more active in fetuin desialylation compared to the N1-VLPs or ΔH1N1-VLPs (Figure 4(C)). Relative NA activities were calculated using the serial dilution curves obtained in NA-Star assay and ELLA (Figure S2). Data obtained from both low pH-treated virions and HA-defective VLPs indicated the enhancement of NA activity when HA is co-expressed and binds to SA receptors.

### The presence of HA enhances NA activities against both O-linked and N-linked glycans

**Figure 5.**
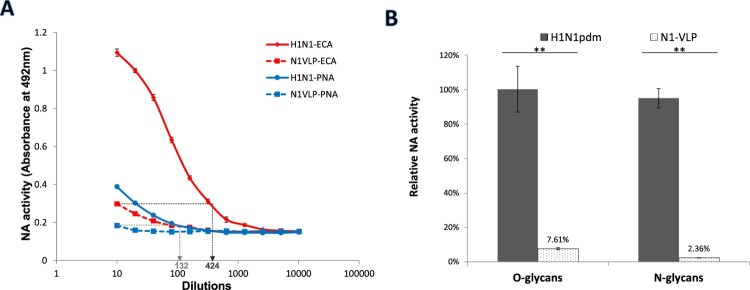
Desialylation of N- and O-linked glycans in fetuin by influenza virus and NA-VLP. NA activities of H1N1pdm virus and N1-VLP were measured in ELLA. (A) Desialylated N-glycans and O-glycans were detected using HRP-conjugated ECA and PNA lectins, respectively. (B) Relative NA activities at dilution 1:20 were calculated using the serial dilution curves obtained from H1N1pdm virus. Mean values of 2 independent experiments were showed. *p < .05; **p < .01.

Fetuin contains sialic acids linked to both O-glycosidically linked and N-glycosidically linked carbohydrate chains [31]. For the detection of NA activities against O-glycans and N-glycans in ELLA, lectins peanut agglutinin (PNA) and *Erythrina cristagalli* (ECA) were used respectively. Using fetuin as the substrate, NA activities against both O-glycans and N-glycans were measured from N1-VLPs and the native H1N1pdm viruses. Although stronger signals were found from desialylated N-glycans than that from O-glycans (Figure 5(A)), higher NA activities were observed in H1N1pdm viruses compared to the N1-VLPs using either lectins. This indicates that NA activities against both O-linked and N-linked glycans were elevated by the presence of HA (Figure 5(B)).

### Effect of HA on NA activity is prominent in immobilized fetuin compared to 3′SLN

**Figure 6.**
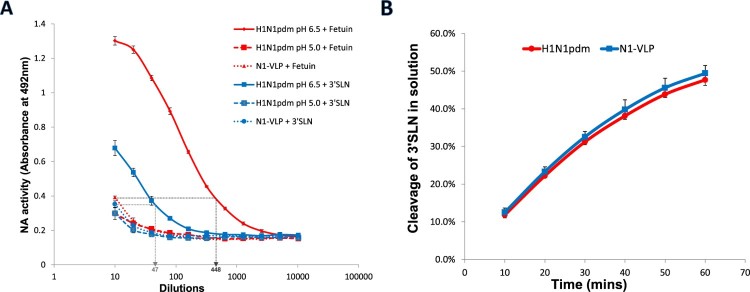
Desialylation of fetuin and 3′SLN by influenza virus and NA-VLP. (A) NA activities of NA-VLP and low pH pre-treated H1N1pdm viruses were measured in ELLA. Desialylation of fetuin and 3′SLN were detected using HRP-conjugated ECA lectin. (B) Cleavage of 3′SLN in solution was evaluated in NMR spectroscopy. 1H NMR spectra were recorded at 10-minute intervals. The yield of cleavage was quantified by the ratio of different chemical shift from the protons associated with NHAc group

It was noted from the data that HA-receptor binding augmented the NA activity in ELLA against an immobilized complex substrate, fetuin, while no significant difference was detected with NA-Star assay when the substrate was a small soluble molecule. To test the contribution of glycan complexities versus glycan immobilization in the solid phase in the enhancement of NA activity, immobilized 3′SLN (3′sialyl-N-acetyllactosamine-BSA) was used in the place of fetuin in the ELLA. While N1-VLPs and acid-treated viruses displayed similar enzymatic activities against 3′SLN and fetuin, control treatment virus desialylated fetuin and 3′SLN with 44.8-folds and 4.7-folds of enhancement, respectively (Figure 6(A)). Sialic acid cleavage against soluble 3′SLN was also assessed using NMR spectroscopy as described [22], in which no significant difference in NA activity was detected (Figure 6(B)). Differential results obtained from the two assays indicated the importance of substrate immobilization in HA-enhanced NA activity; whereas dissimilarity between the cleavage of 3′SLN and fetuin in ELLA suggested a role of glycan structure in this phenomenon.

### Substrate specificity of NA was affected by altered HA-receptor specificity

**Figure 7.**
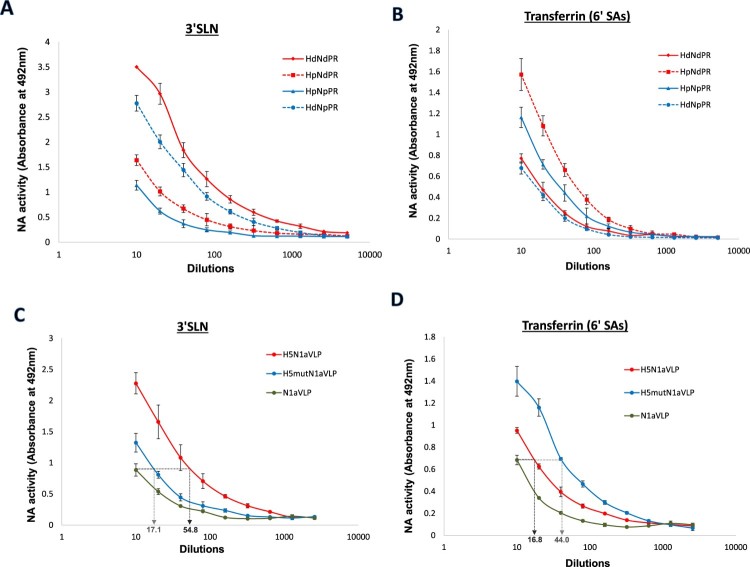
Desialylation of 3′SLN and human transferrin by recombinant H1N1 viruses and H5N1-VLPs. Recombinant H1N1 viruses containing HA and NA from Duck/H1N1 (H_d_, N_d_) or H1N1pdm (H_p_, N_p_) were prepared by reverse genetics. NA activities of the recombinant viruses against (A) 3′SLN-BSA or (B) human transferrin were measured in ELLA. H5N1a-VLP containing HA and NA from H5N1/VN1203 were constructed along with an α2,6 SA-binding mutant (H5_mut_) and the NA activities against (C) 3′SLN-BSA or (D) human transferrin were measured in ELLA. Mean values of 2 independent experiments were showed.

To explore the effect of HA-receptor binding specificity on NA activities against different substrates, reassortant influenza viruses contain combinations of HA and NA from human H1N1pdm and an avian Dk/H1N1 virus were generated by reverse genetics technology. HA of H1N1pdm preferentially binds to α2,6-linked SA found in the human upper airways whereas HA of Dk/H1N1 has restricted binding to α2,3-linked receptors found in avian species [32]. Reassortant viruses were normalized by NA activities in NA-Star assay as described above. Human transferrin and 3′SLN were utilized as “human” and “avian” SA receptors in ELLA. When the HA of H1N1pdm virus was replaced by an avian Dk/H1N1 HA using reverse genetics, higher NA activity against 3′SLN was detected but the activity against human transferrin dropped (Figure 7(A,B), H_p_N_p_ against H_d_N_p_). A reversed phenomenon was found when we changed the HA of the Dk/H1N1 virus to that from H1N1pdm virus (Figure 7(A,B), H_d_N_d_ against H_p_N_d_). These indicated the NA activities against transferrin and 3′SLN were affected by a change in HA-receptor preference.

To provide further evidence that HA-mediated enhancement in NA activity against sialoglycans with different SA linkages can be affected by a switch of HA-receptor preference, H5N1a-VLP containing HA and NA from avian H5N1 virus were constructed. Double mutations Q226L, G228S were introduced in the HA sequence to generate H5_mut_N1a-VLP with a switch of binding specificity from an avian to human receptors [33,34]. N1a-VLPs expressing only the NA from the H5N1 virus were also produced to evaluate the NA enchantment by the presence of HA. VLP samples were normalized in NA-Star assay and NA activities against transferrin and 3′SLN were measured in ELLA (Figure 7(C,D)). Introduction of wildtype α2,3-binding H5 into N1a-VLP led to a higher enhancement of NA activity against 3′SLN (5.48 times) than that against transferrin (1.71 times), whereas the presence of α2,6SA-binding H5_mut_ resulted in higher NA enhancement against transferrin in comparison with 3′SLN (4.4 and 1.68 times, respectively). The above data indicated a correlation between HA-receptor preference and NA specificity against human and avian sialoglycans.

## Discussion

Sialic acid molecules on the host cell surface are regarded as the influenza virus receptors that interact with both HA and NA [3]. It is thus rational that HA and NA may interact in the receptor-recognitions of each other and therefore, on the functionality of each protein. In this study, we investigated the effect of HA-SA binding on NA activity in influenza A virus using both native viruses and VLPs. Throughout the study, NA-containing samples were normalized by their activities in NA-Star assay which reflected the amount of NA content on viral or VLP surface as confirmed by western blot assays (Figures 1(B), 2(A) and 4(A)). Although comparison of purified NAs and their original viruses has been previously described, the reported study utilized soluble substrates to measure NA activities and was aimed to detect any difference in substrate specificity [35]. In the present study, distinctive differences in NA activities between NA-VLPs, HANA-VLPs and the native H1N1pdm virions were found in ELLA indicating that the NA enzymatic activity is enhanced by the presence of HA (Figures 1 and 2). However, such differences in catalytic efficacy were neither detected in NA-Star assay nor in NMR spectroscopy using soluble substrates, suggesting that the HA-mediated enhancement may be limited to surface-anchored sialoglycans. A similar phenomenon has been found in coronavirus in which receptor-destroying function of haemagglutinin-esterase is affected by the lectin binding activity from the same glycoprotein [36].

Treatment of influenza virions with low pH leads to a loss of infectivity due to the irreversible conformational change in HA to the fusogenic state [28]. Herein we attempted to selectively inhibit HA binding to SA receptor using low pH treatment and found that both H1N1pdm and avian H9N2 viruses pre-treated at pH 5.0 lost the ability to agglutinate TRBC (Figure 3). The absence of HA-receptor binding in low pH treated viruses led to much lower NA activities indicating a role of HA-SA binding in assisting the enzymatic function of NA. Like the results obtained using NA-VLPs, a big difference in NA activity was only detected in ELLA in contrast to the NA-Star assay. Experiments using HANA-VLPs and HA-defective mutant (Figure 4) confirmed the hypothesis that HA bindings to sialic acids led to an elevated NA catalytic activity against immobilized substrates in ELLA. Collectively, the above data demonstrated a cooperative interplay between HA and NA functions that may affect the functional balance between the two proteins. A similar conclusion has been drawn by Kosik and Yewdell [20], in their study showing that anti-HA antibodies could inhibit NA activity in ELLA by blocking virus attachment. A haemadsorption site separated from the catalytic site was discovered in the NA of some avian influenza viruses which may also influence the catalytic efficiency [37,38]. Such a secondary SA-binding site is unlikely to play a role in the present study since both NAs from H1N1pdm and H9N2 (HK/G1/97) do not contain a significant haemadsorption site [39,40]. However, it will be interesting to investigate if NA-receptor bindings via the haemadsorption site can lead to a similar enhancement effect on NA catalytic efficiency.

PNA lectin is commonly used in ELLA to bind the exposed terminal galactose during desialylation of carbohydrates mediated by neuraminidase. However, PNA specifically binds to Galβ1-3GalNAc and therefore limits the detection to desialylated O-glycans [41]. N-linked glycans typically consist of Galβ1-4GalNAc residues which are poorly bound with PNA. Therefore an additional lectin such as ECA is necessary for the detection of desialylated N-glycans [42]. The results showed that NA-VLPs and H1N1pdm virus were able to cleave both O-linked and N-linked sialoglycans on fetuin (Figure 5). Although optimal dilutions of lectins were tested and applied in all experiments, PNA (EY Lab) produced lower maximum signals with compared to the PNA (Merck) utilized in standard ELLA protocol (as in Figures 1–4), indicating that the signal intensity in ELLA may vary between lectins from different suppliers or preparations. Using both PNA and ECA lectins from the same supplier, higher activities were detected from the whole virions compared to the NA-VLPs suggesting that the presence of HA enhance NA activities against both types of glycans. Fetuin contains a higher level of N-glycans than O-glycans as estimated by the sugar compositions [43], which may explain the higher signals obtained by ECA than PNA in ELLA.

**Figure 8.**
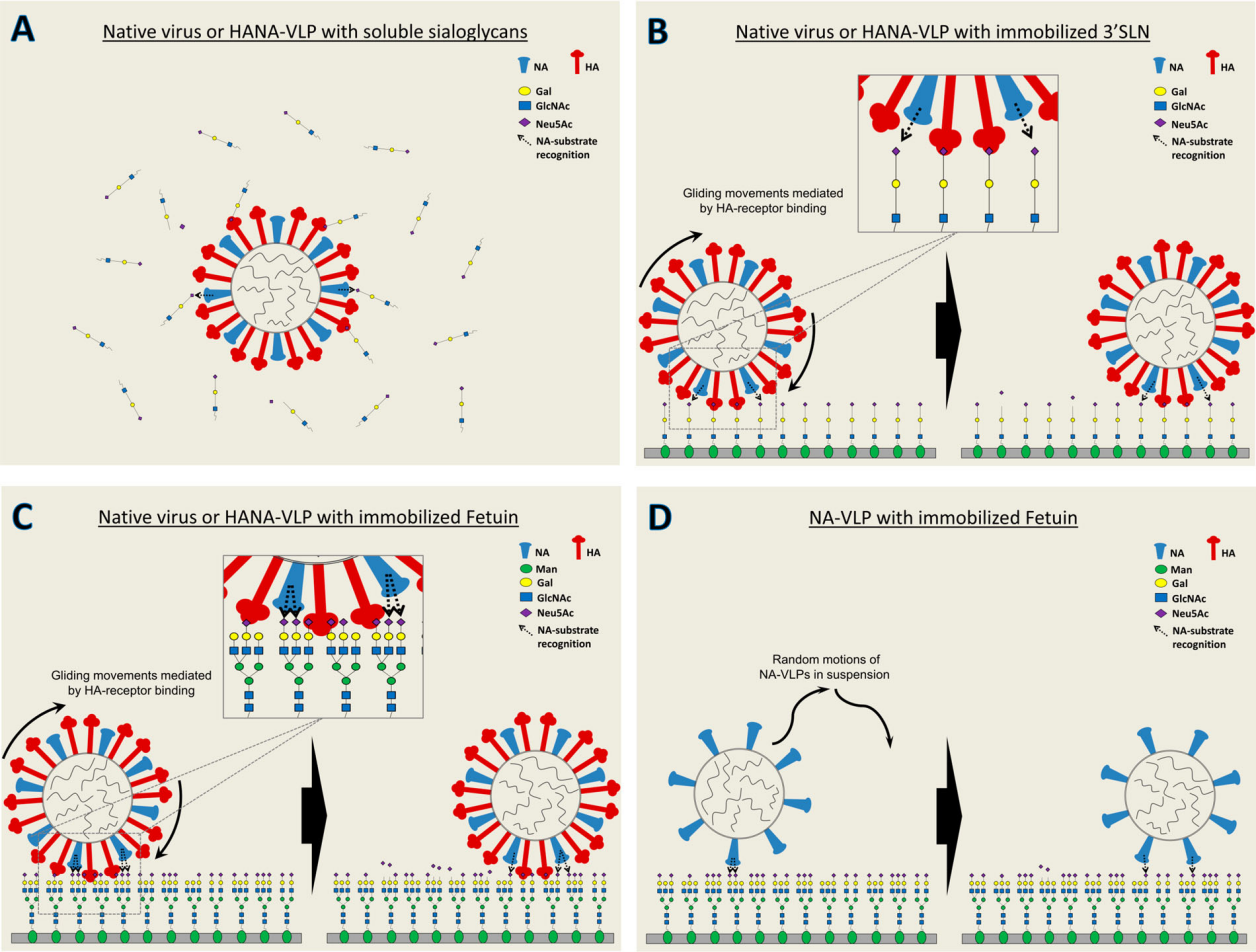
Proposed models of NA-substrate recognition in influenza virions and VLPs.

It is important to note that the co-existing influenza HA led to a strong enhancement of the NA activity against immobilized fetuin, a moderate enhancement to that against immobilized 3′SLN, but not in experiments using soluble substrates (Figure 6). These data suggested that both glycan presentation and glycan complexities contribute to the enhanced catalytic activity. Figure 8 shows a schematic model that explains our experimental findings on the effect of different substrates and HA co-expression on NA activities of the influenza virus or VLPs. In a mixture of viral particles and sialoglycans in suspension, NA recognizes the SA substrates by random motions (Figure 8(A)) therefore no significant difference in NA activities were detected between H1N1pdm viruses and NA-VLPs in NA-Star assay (Figures 1(A) and 3(B)) or NMR spectroscopy (Figure 6(B)). Whereas in ELLA, surface-anchored sialoglycans formed a multivalent receptor surface for the HA bindings which allows the virus or HANA-VLP to stay on the glycan surface and thus promotes NA-substrate recognition and the catalytic efficiency (Figure 8(B,C)). Recent studies demonstrated that HA and NA cooperatively mediate the movements of virions over a receptor-coated surface [18], and the efficient cleavage of sialoglycans can only be detected when there is HA-dependent virus binding to the receptors [19]. High density of HA on the virion surface allows multiple binding events with the receptors. In the absence of NA functions, influenza viruses adhered to the cellular membranes [10]. NA catalytic activity, especially with the HA-mediated enhancement, possibly reduces multivalent binding of virus HA to the cell surface and prevents virus sticking. Simultaneous interactions of HA, NA and SA receptors allows the ‘browsing’ movements of the viruses until they reach the endocytic sites.

In comparison to the use of fetuin, utilizing 3′SLN as the substrate in ELLA led to a lower enhancement in NA activity, indicating an influence of glycan complexity in the enhancement of NA function by HA (Figure 6(A)). Over 95% of the N-glycans on fetuin were found to be biantennary or triantennary glycans that contain multiple sialic acids on single molecules [44]. HA binding to one SA of the bi/tri-antennary glycans may bring the NA in close contact with other SAs on the same molecule and therefore facilitate the NA catalytic efficiency (compare enlarged model in Figure 8(B) and 8(C)). In the absence of HA, NA-VLPs are unable to stay on the glycan surface and therefore only access to the sialic acids by random motions in suspension, which resulted in a lower NA activity (Figure 8(D)). In fact, natural biologic systems including the human respiratory tract contains complex multivalent sialylated glycans [32] and therefore the results obtained in ELLA with fetuin mimic the biological situation more closely than other NA activity assays. Although the significance of the HA-mediated NA activity enhancement in physiological conditions is not known for certain, it is likely to play a role in the penetration through mucus in which multivalent sialic acids are abundant. Our findings also highlight the importance of multivalent receptors with the presence of HA in future studies of NA activity.

Since HA modulates NA activity through SA binding, binding affinity of HA to different sialoglycans may influence the substrate preference of NA. Standard ELLA protocol utilized fetuin as NA substrate to contain SA both with α2,3 and α2,6 linkages [31,45]. In order to study NA activities specifically against α2,3 and α2,6 linked SA, BSA-linked 3′SLN and human transferrin were used, respectively, as the substrates in this study. Transferrin was selected because 6′SLN-BSA is not commercially available. Recombinant virus containing HA and NA from Dk/H1N1 (H_d_N_d_PR) displayed a higher activity against 3′SLN with compared to the virus with both HA and NA from H1N1pdm (H_p_N_p_PR), whereas SA cleavage of transferrin was more efficient in the latter virus (Figure 7(A,B), solid lines). However, a swap of HAs between the two viruses led to a reversed NA specificity (Figure 7(A,B), dotted lines), indicating the NA activities against transferrin and 3′SLN were affected by a change in HA-receptor preference. HA from Dk/H1N1 bound strongly to 3′SLN (with α2,3 linked SA) but not to human transferrin (with α2,6 linked SA), therefore enhanced the enzymatic activities of NA from either avian or human viruses to cleave 3′SLN. In contrast HA from H1N1pdm only bound and enhanced NA activities against human transferrin.

Differential receptor recognition speciﬁcities are found in the HA of different influenza viruses, and a switch in SA binding preference is one of the major determinants for the adaptation of avian influenza in human system [6]. HA of avian influenza viruses predominantly bind to α2-3 sialoglycans found abundantly on epithelial cells in birds, whereas most human influenza HAs preferentially bind to α2-6 sialoglycans, which are found on the epithelium of human upper respiratory tract. In contrast, NA specificity was reported to be relatively consistent among viruses isolated from different hosts. NA of all influenza A subtypes were found to cleave α2-3 linked SA more efficiently [11,12,14], which could not explain how human-adapted viruses are efficiently released from the host cells predominantly express α2-6 receptors. These findings were either obtained using purified NA proteins or based on the detection of NA activity against soluble substrates, therefore, may only reflect the NA properties without the interference of HA. Our results in ELLA using recombinant viruses and H5N1VLPs showed that NA specificity against multivalent SAs is affected by the specificity of HA in whole virions. HA of human influenza viruses were able to enhance the cleavage of α2-6 linked SA by the NA from avian isolates. A similar phenomenon may be applied in the avian-to-human host adaptation of the influenza virus to allow the cleavage of sialic acids with either α2-3 or α2-6 linkage, in which NAs retain the specificity against α2-3 SA and the HAs with human-receptor binding preference enhance the NA activity against α2-6 SA. However from the data presented, we cannot conclude whether a change of HA-receptor preference led to a switch of NA specificity due to the dissimilar glycan structures in SLN and transferrin used in this study. An alternative protocol using substrates with identical underlying sugar structure, but different sialic acid linkages will be necessary for a systematic study of NA specificity of influenza viruses in the presence of HA.

In conclusion, the data in this study demonstrated interplay between HA and NA functions in influenza viruses. Binding of HA to SA receptors promotes the sialidase activity of NA especially with immobilized multivalent substrates that mimic the biologic situations where intact virions are confronted with multivalent membrane receptors. We also found that NA activities against different substrates can be affected by the alteration of HA-receptor specificity between avian-like and human-like receptors. Our findings provide new insight into influenza HA/NA functional balance and viral host-adaptation, although the biological significance has yet to be tested. The methodology of low pH incubation for HA-inactivation may also become a useful tool for future study of NA on the whole virus without an interference from the HA functions.

## Materials and methods

### Cells and viruses

MDCK and HEK-293 T cells were cultured in Dulbecco’s modified Eagle’s medium (DMEM) supplemented with 10% foetal bovine serum (FBS) and 1% penicillin/streptomycin at 37°C with 5% CO_2._ Influenza viruses A/California/4/2009 (H1N1pdm) and A/Quail/Hong Kong/G1/97 (H9N2) were propagated in MDCK cells and embryonated chicken eggs respectively. Reassortant H1N1 viruses containing HA and NA segments from H1N1pdm or A/Duck/Bavaria/1/1977 (Dk/H1N1), with the remaining 6 gene segments derived from A/Puerto Rico/8/1934 (PR8/H1N1) virus, were generated by plasmid-based reverse genetics [46] and were propagated in MDCK cells.

### Plasmids

Expression plasmids containing HA or NA were constructed with DNA sequences corresponding to HA (GeneBank accessions: GQ117044(H1N1pdm); EF541403(H5N1/VN1203)) and NA (GeneBank accessions: FJ969517(H1N1pdm); EF541467(H5N1/VN1203)) cloned into mammalian expression vector pcDNA3.1 (Invitrogen). Both NA and HA proteins were FLAG-tagged at the C-terminus. Site-directed mutagenesis was applied for the constructions of HA-defective mutant at RBS of H1N1pdm (ΔH1) with L194A and Y195F amino acid substitutions, and α2,6 SA-binding mutant of H5 (H5_mut_) containing Q226L and G228S double mutations in amino acid sequence.

### Preparation of influenza VLPs

HEK-293 T cells were transfected in 150 mm culture dish with pcDNA-N1 and pcDNA-H1 plasmids using CalPhos Mammalian Transfection kit (Clontech) according to the manufacturer’s instructions. Different DNA plasmid ratios (HA/NA: 0/1, 1/1 and 4/1) were used to produce NA-VLPs containing different level of HA (N1-VLP, H1N1-VLP and H1_high_N1-VLP). At 12 h of post-transfection, the medium was replaced with fresh CD OptiCHO medium (Gibco) and the supernatant was collected at 60 h of post-transfection followed by filtration to remove the cell debris. The filtered culture medium was layered onto a 20% sucrose cushion and centrifuged at 28,000 rpm. for 2.5 h at 4°C, and the VLP pellet were re-suspended in DMEM with 20 mM HEPES. The same protocol was applied to produce ΔH1N1-VLP, H5N1a-VLP and H5_mut_N1a-VLP.

### Antibodies and Western blotting

Native H1N1pdm viruses were detected using antisera from rabbits hyper-immunized with NA-VLPs, followed by goat anti-Rabbit IgG HRP-conjugated secondary antibody (Thermo Fisher). HRP-conjugated anti-FLAG M2 monoclonal antibody (Merck) was used for the detection of FLAG-tagged HA and NA proteins on VLPs. For western blotting, samples were denatured by heating at 95°C for 10 min in LDS sample buffer (Invitrogen) with DTT (100 mM) and were resolved in SDS-PAGE, followed by electroblotting onto PVDF membrane. The PVDF membranes were hybridized with the corresponding antibodies and detected using ECL Select WB Detection Reagent (GE Healthcare). The relative amounts of protein in each band were determined by densitometry using ImageJ (NIH).

### Detection of NA enzymatic activities

The enzymatic (sialidase) activity of influenza VLPs or whole viruses was measured using NA-Star neuraminidase detection kit (Applied Biosystems) or ELLA. NA-Star assays were performed following the manufacturer’s protocol and data were measured in duplicate by MicroBeta luminescence counter (PerkinElmer). ELLA was performed as described previously [47] with a substrate or lectin substitutions in corresponding experiments. Briefly, fetuin (25 µg/ml), 3'SLN-BSA (10 µg/ml; Dextra) or human transferrin (25 µg/ml; Merck) were coated on Maxisorp Nunc 96-well plates (Thermo Fisher Scientific). Sialic acid cleavage by the influenza viruses or VLPs was performed in dilution buffer (MES, pH 6.5 with 20 mM CaCl2, 1% bovine serum albumin and 0.5% Tween 20) at 37°C for 18 h. Plates were washed six times and terminal galactose moieties were quantified using HRP-conjugated peanut agglutinin (PNA; Merck and EY Laboratories) or *E. Cristagalli* (ECA; EY Laboratories) lectins (2 µg/ml final concentration) followed by O-phenylenediamine dihydrochloride (OPD). The plates were read at 492 nm for 0.1S using FLUOstar OPTIMA 96-well plate reader (BMG Labtech). All results were calculated as the means from two or three independent experiments.

Cleavage of 3′SLN in solution was detected using NMR spectroscopy as previously described [22]. Virus and VLP samples were reconstituted in HEPES-d18 buffered saline and 1 mM 3′SLN-BSA was added. ^1^H NMR spectra were acquired at 10-min intervals with 64 scans over a spectral width of 6,000 Hz using a Varian 700 MHz NMR System.

### pH treatments of influenza virus and HA assay

Influenza viruses (H1N1pdm or H9N2) were incubated in 0.1 M acetate buffer (pH 5.0 and 6.5) at 37°C for 1 h and were neutralized to pH 6.5 by the addition of HEPES buffer. The buffer was then exchanged to phosphate buffered saline (PBS, pH 7.4) through Amicon centrifugal filter unit (Millipore). HA assays were performed in 2-folds dilution using 0.5% TRBC on V-bottom 96-well plates.
